# Congenital Junctional Ectopic Tachycardia: Presentation And Outcome

**Published:** 2003-07-01

**Authors:** Berardo Sarubbi, Pasquale Vergara, Michele D'Alto, Raffaele Calabro

**Affiliations:** Second University of Naples, Chair of Cardiology, Division of Paediatric Cardiology, Monaldi Hospital

## Introduction

Junctional ectopic tachycardia (JET) is a rare type of supraventricular arrhythmia. Even if its management has improved in recent years, it remains a great challenge for the cardiologist. Two are the possible clinical presentations of this arrhythmia: as a primary idiopathic disorder during infancy, configuring the so called "congenital" JET, or more often as a transient phenomenon immediately after surgery for congenital heart disease, giving rise to the "post-operative" variety.

The congenital form, firstly described as a distinct entity by Coumel et al. in 1976 [1], usually occurs in the first six months of life presenting as a persistent sustained form, lasting up to 90% of the time. Its clinical presentation may be dramatic, being associated in up to 60% of cases with cardiomegaly and/or heart failure.

Congenital JET is hampered by high mortality. Secondary dilated cardiomyopathy, ventricular fibrillation and sudden cardiac death have also been reported [2,3].

## Clinical Presentation

Clinical presentation of congenital JET usually ranges from birth to 4 weeks of age. Sporadic cases of history of intrauterine tachycardia, with sometimes hydrops fetalis, have been reported in patients who at birth showed overt JET [2,4,5].

Usually, high percentage of patients present, at the time of referring, echocardiographyc evidences of impaired left ventricle function and clinical signs of congestive heart failure. However, it has been noted that age of tachycardia presentation is not related to the occurrence of congestive heart failure or impaired left ventricular function. On the contrary, clinical status seems related to the ventricular rate at presentation [6].

The incidence of congenital heart disease in presence of congenital junctional ectopic tachycardia has been reported sporadic [6].

## Diagnostic Assessment

**Figure 1:**
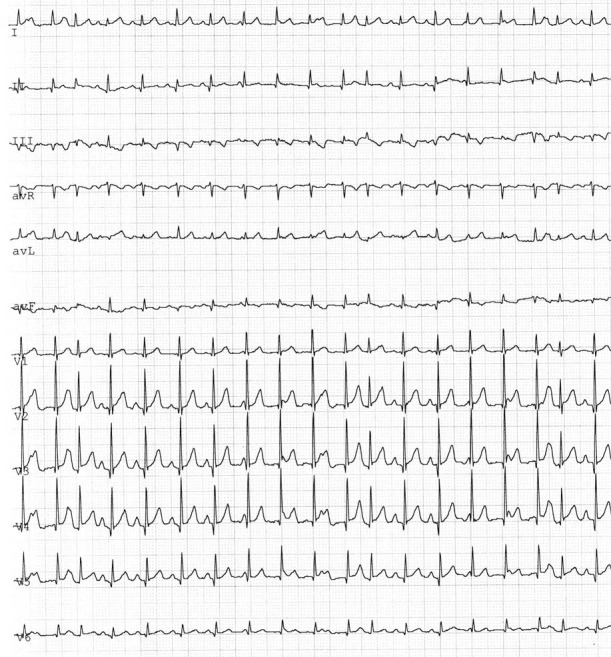
Junctional ectopic tachycardia : a narrow complex tachycardia with atrio-ventricular dissociation

The diagnosis of junctional ectopic tachycardia usually is based on electrocardiographic evidence of a narrow complex tachycardia (heart rate ranging from 160 bpm to 300 bpm) and atrio-ventricular dissociation. (Figure 1) Sometimes, it is possible to observe slight variation of heart rate during the 24 hours and irregularity in the RR due to sinus capture beats. Distinct P waves can be seen only in the tracing of the patients with slower heart rate following drug treatment.

Prenatal diagnosis of JET has been also reported, with the superior vena cava/ascending aorta Doppler echocardiography approach [4]. Doppler wave showed AV dissociation with aortic ejection occurring at smaller interval than venous retrograde flow due to atrial contractions and "a" waves occurring at a very short interval after aortic ejection, during periods of 1:1 AV relationship tachycardia.

## Electrophysiological Mechanism

Electrophysiological mechanism of JET is thought to be abnormal automaticity within the His bundle [7]. JET typically presents a "warming-up" phase at its onset and a "cooling-down" phase at its termination. It is usually unresponsive to overdrive pacing or DC shock. When there is retrograde conduction, adenosine infusion can rule out the diagnosis of AV reciprocating tachycardia, because of tachycardia cycle lengthening without termination of the arrhythmia [8].

Tachycardia is not inducible by programmed stimulation in baseline status, but can be elicited after sympathetic stimulation with isoproterenol. Programmed atrial and ventricular stimulation usually are not able to terminate tachycardia, that can be only transiently suppressed by atrial and/or ventricular pacing, exhibiting a resetting curve-response to extrastimuli during tachycardia.

JET can coexist with the occurrence of other types of arrhythmias. Scheinman et al. [9] reported the development of JET after catheter ablation of persistent junctional reciprocating tachycardia in one patient, but a differentiation between a masking effect of the latter tachycardia on JET and a procedure related genesis of JET itself could not be argued.

## Familiar occurrence

Even more interest is attributed to this arrhythmia by the fact that it is a familiar condition. In a multicenter study, Villain reports a familiar history of JET positive in 50% of the children. [2]. In our experience up to three members of the same family (2 sisters, 1 cousin) were affected by JET with a family history of JET recognised in up to 55.6% of the patients [6]. These characteristics suggest a possible Mendelian inheritance but, at the moment, a specific gene has not been identified.

## Outcome

Congenital JET is hampered by high mortality, with up to 34% mortality rate. The exact mechanism related to death in congenital JET is still unclear. Most are sudden death, that has been found also in children with "well compensated" tachycardia [2].

Sporadic case of sudden death has been attributed to a dramatic evolution to paroxysmal complete AV block (2; 10). Probably, the dramatic clinical course of these patients is the extreme expression of the pathological process occurring in the His bundle region, causing JET in a first time and finally the complete loss of AV conduction.

Necropsy reports underlined the importance of His-bundle fibbers severe disruption [11,12] or focal degeneration and split2. In other cases the arrhythmia has been attributed to extensive abnormalities involving the whole atrio-ventricular junction [13] or to an intra-His bundle tumour originating from Purkinje cells [14].

Henneveld reported a case of an 8 months old girl affected by a rapid ventricular rate JET, who developed two years later a complete heart block with rapidly increasing left ventricular end diastolic diameter [10]. The patient was not assuming therapy at the time of the onset of complete heart block.

In other cases it can not be excluded that a pharmacological treatment can be responsible of the depression of ventricular excitability, leading to a slow unsatisfactory ventricular escape rhythm in case of AV block and sudden cardiac death. However, in some cases it can not be excluded the potential proarrhythmic effect of the drugs used to control the arrhythmia [15]. In our series, two patients developed symptomatic non-sustained life-threatening ventricular tachycardia as a spontaneous desincronization of junctional ectopic tachycardia, due to a proarrhythmic effect of the combination of used drugs (Amiodarone + Propafenone and Amiodarone + Flecainide) [6].

## Pharmacological Treatment

Treatment is indicated in infants with symptoms, reduced ventricular function or rapid rates [16'^17^]. The management of infants with slow JET (less than 150 beats/min) without symptoms appear to be debated. However, necessity to monitor accurately these asymptomatic patients is undoubted. It has been reported a case in which an asymptomatic patient at birth with junctional ectopic tachycardia with a ventricular rate of 140 beats/min returned at 6 weeks of age in cardiovascular collapse due to a ventricular rate of 300 beats/min [2].

The pharmacological treatment of JET is hampered by a high rate of failures. Several drugs have been tested, but a comprehensive analysis of efficacy has not been performed yet.

The largest study available is a multicenter study performed in nineties by European and American teams who reviewed dates of 26 pts treated with digoxin, propranolol and/or amiodarone [2]. The largest single centre experience of pharmacological treatment has been recently reported, with the use of Amiodarone, Flecainide or Propafenone alone or in combination [6].

Digoxin did not affect ventricular rate in any patients and has been demonstrated to be not completely safe in patients with congenital JET. Sporadic cases have been reported in which patients affected by congenital JET and severe cardiac failure developed ventricular fibrillation or faster tachycardia (up to 400 beats/min) during progressive digoxin loading [2].

Amiodarone seems to have the highest response rates: it has been shown to be effective alone in decreasing the ventricular rate to less than 150 beats/min in a percentage between 50-70% of the cases [2,6].

Sporadic reports of medical treatment efficacy exist also about Propafenone [6,18], Flecainide or Encainide [6,19] and Sotalol [20]. However, Propafenone has been shown to result particularly effective in preventing or controlling the tachycardia only in patients with lower heart rate [6]

Other studies have also tested phenytoin, which was able to control ventricular rate but caused ataxia, ajmaline, which intravenous infusion was followed by ventricular tachycardia and verapamil which caused cardiovascular collapse [2].

Personal experience has shown that in those cases not responding to a single drug regimen, as association of antiarrhythmic agents with different electrophysiological effects (Amiodarone + a Ic antiarrhythmic drug) may control otherwise untreatable congenital JET. Otherwise the association Propafenone or Flecainide plus Amiodarone could reduce the Amiodarone dose keeping an high efficacy of the pharmacological therapy [6].

Recently Dorman et al. have described a protective effect of magnesium supplementation on JET in paediatric patients undergoing surgery for congenital heart defects [21]. This could be due to the stabilization of the membrane potential and the reduction of the automaticity, resulting in a low rate of development of the arrhythmia. Further studies are needed to evaluate the potential role of magnesium in the treatment of the congenital form of JET.

All pharmacological therapies are burdened by a high risk of toxicity as they have to be maintained at high dosage for long time in young patients.

Congestive heart failure is a frequent complication of JET with high ventricular rate and its management is even more difficult. In these cases it has been underlined the negative inotropic effects of antiarrhythmic drugs and the limitations in the use of sympathomimetic agents. Isoproterenol, dopamine, dobutamine and amrinone all increase the JET rate. Sodium Nitroprusside has to be avoided too, since low blood pressure will reflexly increase adrenergic tone.

## Non pharmacological treatment

A definitive treatment could be the removal of the arrhythmogenic area. Surgical His ablation has been attempted in some critical patients, with contrasting results [2]. In one case cautery surgical ablation led to the development of ventricular tachycardia. Radiofrequency catheter ablation of JET was reported for the first time in the eighties [22,23]. It required, in the first procedures, the elimination of AV conduction tissue with the following pacemaker implantation. In nineties radiofrequency catheter ablation technique was able to control the tachycardia with the preservation of the normal AV conduction [9]. Lesions were carried out in the region between the coronary sinus and the anterior septum, moving progressively anteriorly to a site where a small His deflection was recorded. In some cases His-bundle localization can be uncommon, in view of the fact that a His-bundle has been found on the left sided of the interventricular septum [2]. Recent experiences have proven the feasibility of the selective modification of the AV junction without the complete destruction of the conduction system even in small children, less than one year old [24].

Very controversial is still the matter of prophylactic pacemaker implant to prevent sudden death. In the multicenter study [2] most part of patients did not undergo to pacemaker implantation, but four patients with well compensated JET died suddenly and in one of those a slow ventricular rate was recorded during the terminal event.

Walsh suggested to evaluate AV conduction with transesophageal electrophysiological test in all patients with congenital JET and consider pacemaker insertion only if impaired conduction can be demonstrated by atrial stimulation, or by the observation of spontaneous AV block on ECG and Holter monitoring [25].

## Conclusions

Since JET therapy relay on different medical options, a staged treatment protocol is advisable: a pharmacological approach should be tested and carried on, also with a multi drug treatment, while catheter ablation can be a definitive option in critically ill patients, when drugs fail or when tachycardia becomes chronic and the patient is dependent on a potentially toxic therapy.

## References

[R1] Coumel P, Fidelle JE, Attuel P (1976). Tachycardies focales hisiennes congenitales: etude cooperative de sept cas. Arch. Mal. Coeur.

[R2] Villain E, Vetter VL, Garcia JM (1990). Evolving concepts in the management of congenital junctional ectopic tachycardia. A multicenter study. Circulation.

[R3]  Brechenmacher C, Coumel P, James TN (1976). Intractable tachicardia in infancy. Circulation.

[R4] Villazon E, Fouron JC, Fournier A (2001). Prenatal diagnosis of junctional ectopic tachycardia. Pediatr Cardiol.

[R5] Benito Bartolome F, Jimenez Casso S (2000). Hydrops fetalis secondary to junctional ectopic tachycardia. Rev. Esp. Cardiol.

[R6] Sarubbi B, Musto B, Ducceschi V (2002). Congenital junctional ectopic tachycardia in children and adolescents: a 20 year experience based study. Heart.

[R7] Garson W, Gillette PC (1979). Junctional ectopic tachycardia in children: electrocardiography, electrophysiology and pharmacological response. Am J Cardiol.

[R8] Rossi AF, Kipel G, Golinko RJ (1991). Use of adenosine in postoperative junctional ectopic tachycardia with 1:1 retrograde atrial conduction. Am Heart J.

[R9] Scheinman MM, Gonzalez RP, Cooper MW (1994). Clinical and electrophysiologic features and role of catheter ablation techniques in adult patients with automatic atrioventricular junctional tachycardia. Am J Cardiol.

[R10] Henneveld H, Hutter P, Bink-Boelkens M (1998). Junctional ectopic tachycardia evolving into complete heart block. Heart.

[R11] Brechenmacher C, Coumel P, James TN (1976). Intractable tachycardia in infancy. Circulation.

[R12] Till JA, Ho SY, Rowland E (1992). Histopathological findings in three children with His bundle tachycardia occurring subsequent to cardiac surgery. Eur Heart J.

[R13] Bharati S, Moskowitz WB, Scheinman M (1991). Junctional tachycardias: anatomic substrate and its significance in ablative procedures. J Am Coll Cardiol.

[R14] Rossi L, Piffer R, Turolla E (1985). Multifocal Purkinje like tumour of the heart: occurrence with other anatomic abnormalities in the atrioventricular junction of an infant with junctional tachycardia, Lown-Ganong-Levine syndrome and sudden death. Chest.

[R15] Yap SC, Hoomtje T, Sreeram N (2000). Polymorphic ventricular tachycardia after use of intravenous amiodarone for postoperative junctional tachycardia. Int J Cardiol.

[R16] Wren C (1998). Incessant tachycardias. Eur Heart J.

[R17] Walsh EP, Saul P, Sholler GP (1997). Evaluation of a staged treatment protocol for rapid automatic junctional tachycardia after operation for congenital heart disease. J Am Coll Cardiol.

[R18] Paul T, Reimer A, Janousek J (1992). Efficacy and safety of Propafenone in congenital junctional ectopic tachycardia. J Am Coll Cardiol.

[R19] Kuck KH, Kunze KP, Schluter M (1988). Encainide versus flecainide for chronical atrial and junctional ectopic tachycardia. Am J Cardiol.

[R20] Maragnes P, Fournier A, Davignon A (1992). Usefulness of oral sotalol for the treatment of junctional ectopic tachycardia. International Journal of Cardiology.

[R21] Dorman BH, Sade RM, Burnette JS (2000). Magnesium supplementation in the prevention of arrhythmias in pediatric patients undergoing surgery for congenital heart defects. Am Heart J.

[R22] Gallagher JJ, Svenson RH, Kasell JH (1982). Catheter technique for closet chest ablation of the atrioventricular conduction system. A therapeutic alternative for the treatment of supraventricular tachycardia. N Engl J Med.

[R23] Gillette PC, Garson A, Porter CJ (1983). Junctional automatic ectopic tachicardia: new proposed treatment by transcatheter His bundle ablation. Am Heart J.

[R24] Fishberger SB, Rossi AF, Messina JJ (1998). Successful radiofrequency catheter ablation of congenital junctional ectopic tachycardia with preservation of atrioventricular conduction in a 9-month-old infant. Pacing Clin Electrophysiol.

[R25] Walsh EP, Saul JP, Triedman JK (2001). Cardiac arrhythmias in children and young adults with congenital heart disease.

